# Cytotoxicity of the methanol extracts of *Elephantopus mollis*, *Kalanchoe crenata* and 4 other Cameroonian medicinal plants towards human carcinoma cells

**DOI:** 10.1186/s12906-017-1793-1

**Published:** 2017-05-25

**Authors:** Victor Kuete, Fabrice W. Fokou, Oğuzhan Karaosmanoğlu, Veronique P. Beng, Hülya Sivas

**Affiliations:** 1 Department of Biochemistry, Faculty of Science, University of Dschang, Dschang, Cameroon; 2 Department of Biochemistry, Faculty of Science, University of Yaounde I, Yaounde, Cameroon; 3 Department of Biology, Science Faculty, Anadolu University, Eskişehir, Turkey

**Keywords:** Cameroon, Carcinoma, Cytotoxicity, *Elephantopus mollis*, *Kalanchoe crenata*, Mode of action

## Abstract

**Background:**

Cancer still constitutes one of the major health concerns globally, causing serious threats on patients, their families, and the healthcare system.

**Methods:**

In this study, the cytotoxicity of the methanol extract of *Elephantopus mollis* whole plant (EMW), *Enantia chlorantha* bark (ECB), *Kalanchoe crenata* leaves (KCL), *Lophira alata* bark (LAB), *Millettia macrophylla* leaves (MML) and *Phragmanthera capitata* leaves (PCL) towards five human solid cancer cell lines and normal CRL2120 fibroblasts, was evaluated. Extracts were subjected to qualitative chemical screening of their secondary metabolite contents using standard methods. The cytotoxicity of samples was evaluated using neutral red uptake (NR) assay meanwhile caspase activation was detected by caspase-Glo assay. Flow cytometry was used to analyze the cell cycle distribution and the mitochondrial membrane potential (MMP) whilst spectrophotometry was used to measure the levels of reactive oxygen species (ROS).

**Results:**

Phytochemical analysis revealed the presence of polyphenols, triterpenes and sterols in all extracts. The IC_50_ values of the best samples ranged from 3.29 μg/mL (towards DLD-1 colorectal adenocarcinoma cells) to 24.38 μg/mL (against small lung cancer A549 cells) for EMW, from 2.33 μg/mL (mesothelioma SPC212 cells) to 28.96 μg/mL (HepG2 hepatocarcinoma) for KCL, and from 0.04 μg/mL (towards SPC212 cells) to 0.55 μg/mL (towards A549 cells) for doxorubicin. EMW induced apoptosis in MCF-7 cells mediated by MMP loss and increased ROS production whilst KCL induced apoptosis via ROS production.

**Conclusion:**

This study provides evidences of the cytotoxicity of the tested plant extract and highlights the good activity of *Elephantopus mollis* and *Kalanchoe crenata.* They deserve more exploration to develop novel cytotoxic drugs.

## Background

Cancer still constitutes a major health concern globally, causing serious threats on patients, their families, and the healthcare system. The related economic impact is significant and is increasing, with annual cost in 2010 being estimated at about 1.16 trillion US dollars [1]. About 70% of deaths caused by cancer occur in low- and middle-income countries. Chemotherapy is recognized as the major mode of treatment of malignant diseases, and the plant kingdom has been the origin of many cytotoxic drugs such as paclitaxel (from *Taxus brevifolia*) and *Vinca* alkaloids (from *Catharanthus roseus*) [2–5]. The potential of African flora as a source of a variety of cytotoxic agents is intensively being demonstrated [6, 7]. In fact, various cytotoxic plants of the continent were reported amongst which are *Anthocleista schweinfurthii, Morus mesozygia, Nauclea latifolia, Erythrina sigmoidea* [8], *Erythrina sacleuxii*, *Albizia gummifera, Strychnos usambarensis, Zanthoxylum gilletii, Bridelia micrantha, Croton sylvaticus, Albizia schimperiana, Erythrina burttii, Erythrina sacleuxii*, *Bridelia micarantha*, *Zanthoxylum giletii* and *Solanum aculeastrum* [9]. In our continuous search for cytotoxic agents from African flora, this study was undertaken to evaluate the antiproliferative activity of the methanol extracts of six Cameroonian plants used traditionally to treat cancers or disease states with symptoms related to cancer. These plants included *Enantia chlorantha* Oliv. (Annonaceae), *Elephantopus mollis* Kunth (Asteraceae), *Kalanchoe crenata* (Andrews) Haworth (Crassulaceae), *Lophira alata* Banks ex C.F.Gaertn*.*(Ochnaceae), *Millettia macrophylla* Benth. (Fabaceae) and *Phragmanthera capitata* (Spreng.) Balle (Loranthaceae). The study was extended to the assessment of the mode of action of the best extracts, namely those from *Elephantopus mollis* whole plant (EMW) and *Kalanchoe crenata* leaves (KCL).

## Methods

### Plant material and extraction

Plants studied in this work are used in the traditional medicine to treat cancer or disease states with symptoms related to cancer (Table 1). They were collected in different parts of Cameroon in February 2015 and included barks of *Lophira alata* and *Enantia chlorantha*, leaves of *Phragmanthera capitata*, *Kalanchoe crenata* and *Millettia macrophylla* and the whole plant of *Elephantopus mollis*. The identification of palnts was done by the Cameroon National Herbarium (HNC; Yaounde) and voucher specimens are availaible under accession numbers (Table 1). The powder obtained from each air dried plant sample (300 g) was macerated in methanol (MeOH, 1 L) for 48 h at room temperature. The macerate was further concentrated under reduced pressure to obtain the crude extract. All extracts were then conserved at 4 °C.

**Table 1:** Published bioactivity and phytochemistry of the studied plants

Species (family); Voucher Number^a^	Traditional uses	Parts used (%yield)^b^	Phytochemical composition (This study)	Bioactive or potentially bioactive components	Bioactivity of crude extract^c^
*Enantia chlorantha* Oliv. (Annonaceae)/ 32,065/HNC	Treatment of Rickettsia fever, cough and wounds, typhoid fever and infective hepatitis or jaundice, urinary tract infections [30, 31]	Bark (3.58%)	Alkaloids, flavonoids, saponins, triterpenes, tannins, steroids, polyphenols	Alkaloids, saponins, cardiac glycosides [31]	Antipyretic [32], antimicrobial and antimalarial activities [33]
*Elephantopus mollis Kunth* (Asteraceae)/ 39,570/HNC	Treatment of various types of cancers and liver infections [25], cough, anemia, dysentery, hepatitis and a number of free radical-mediated diseases including cancer and diabetes [34]	Whole plant (1.77%)	Flavonoids, steroids, triterpenes, polyphenols	Sesquiterpene lactones [25]; 3,4-di-*O*-caffeoyl quinic acid [34]; 28Nor-22(R)Witha 2,6,23-trienolide [28]	Cytotoxic of ethyl acetate extract towards HepG2 cells with the lowest EC_50_ value of 9.38 μg/mL, NCI-H23 cells (13.17 μg/mL), T-47D cells (12.57) and Caov-3 cells (42.11 μg/mL) [25]; antidiabetes activity [28]
*Kalanchoe crenata* (Andrews) Haworth (Crassulaceae)/ 35,196/HNC	Used as antidiabetic and anti-inflammatory drug [35]; Treatment of headache, general debility, dysentery, smallpox and convulsion [36]	Leaves (5.06%)	Alkaloids, steroids, tannins, saponins, flavonoids, polyphenols, triterpenes	Terpenoids, tannins, polysaccharids, saponins, flavonoids and alkaloids [37]	Antimicrobial activity [36]; analgesic and anticonvulsant effects [38] and antihyperglycaemic [37]
*Lophira alata* Banks ex C.F.Gaertn*.*(Ochnaceae)/44,073/HNC	Treatment of febrile conditions, cough, jaundice, and gastrointestinaldisorders [39]	Bark (7.13%)	Alkaloids, steroids, saponins, flavonoids, polyphenols, triterpenes	Lophirachalcone, alatachalcone [40]; lophirone L, lophirone M, luteolin and lithospermoside [41]; isoflavonoids [42]	Cytotoxic, antimutagenic, and antioxidant activities of methanolic extract and chalcone dimers (lophirones B and C) [29]; Lophirachalcone, alatachalcone showed anti-inflammatory activity and inhibited tumor promotion caused by 12-*O*-tetradecanoylphorbol-13-acetate [40]
*Millettia macrophylla* Benth. (Fabaceae)/ 24,038/HNC	Treatment of respiratory difficulties, constipation, colds and headaches, jaundice, cancer as well as some physiological disorders related to menopause [26]	Leaves (4.20%)	Alkaloids, flavonoids, saponins, tannins, steroids, polyphenols, triterpenes	Lupenone, lupeol, stigmastenone, palmitic acid, daidzein dimethylether, formononetin, afromorsin, secundiferol I, 2′-hydroxyformononetin, pisatin, flemichapparin B, dihydrocoumestrol dimethyl ether and variabilin [26]	Estrogenic effect [26]; poor cytotoxic effects of compounds and phenolic fractions towards breast cancer cells MCF-7 and MDA-MB-231 [26]
*Phragmanthera capitata* (Spreng.) Balle (Loranthaceae)/ 24,667/HNC	Treatment of fever, diabetes, abdominal pains, parasitic diseases, and urinary tract infections [43]	Leaves (15.44%)	Alkaloids, flavonoids, saponins, tannins, steroids, polyphenols, triterpenes	Lactones, rel-(1R,5S,7S)-7-[2-(4-hydroxyphenyl)ethyl]-2,6-dioxabicyclo[3.3.1]nonan-3-one and 4-{2-[rel-(1*R*,3*R*,5*S*)-7-oxo-2,6-dioxabicyclo[3.3.1]non-3-yl]ethyl}phenyl 3,4,5-trihydroxybenzoate; betulinic acid, dodoneine, quercetin 3-*O*-*α*-_L_-rhamnopyranoside, quercetin 3-*O-α*-L-arabinofuranoside, quercetin, betulin, lupeol and sitosterol [43]	Antiviral effects against hepatitis C virus [44]; antiplasmodial activity [43]

^a^(HNC): Cameroon National Herbarium; ^b^ yield calculated as the ratio of the mass of the obtained methanol extract/mass of the plant powder; Underline: disease states bearing relevance to cancer or cancer-like symptoms; ^c^Cell line [HepG2: hepatocarcinoma cells; NCI-H23: lung cancer cells; T-47D, MCF-7 and MDA-MB-231: breast cancer cells; Caov-3: ovarian cancer cells]

### Phytochemical investigations

Various classes of secondary metabolites including anthraquinones (Borntrager’s test), alkaloids (Dragendorff’s and Mayer’s tests), coumarins (Lacton test), flavonoids (Aluminum chloride test), polyphenols (Ferric chloride test), saponins (Foam test), sterols (Salkowski’s test), triterpenes (Libermann Burchard’s test) and tannins (Gelatin test) were detected using described phytochemical methods [10–13].

### Chemicals

The reference drug used in this work was doxorubicin 98.0%, purchased from Sigma-Aldrich (Munich, Germany).

### Cell lines and culture

Five carcinoma and one normal cell lines were tested in this work. They were SPC212 human mesothelioma cell line obtained from American Type Culture Collection (ATCC) and provided by Dr. Asuman Demiroğlu Zergeroğlu (Gebze Technical University, Kocaeli, Turkey), A549 human non-small cell lung cancer (NSCLC) cell line, obtained from the Institute for Fermentation, Osaka (IFO, Japan) and provided by Prof. Dr. Tansu Koparal (Anadolu University, Eskisehir, Turkey), HepG2 hepatocarcinoma cells obtained from ATCC and MCF-7 breast adenocarcinoma cells obtained from ATCC and provided by Prof. Dr. Tansu Koparal (Anadolu University, Eskisehir, Turkey), DLD-1 colorectal adenocarcinoma cell lines obtained from ATCC and the normal CRL2120 human skin fibroblasts obtained from ATCC. The cells were maintained as a monolayer in DMEM medium (Sigma-aldrich, Munich, Germany), supplemented with 10% fetal calf serum and 1% penicillin (100 U/mL)-streptomycin (100 μg/mL) in a humidified 5% CO_2_ atmosphere at 37 °C.

### Neutral red (NR) uptake assay

The cytotoxicity of samples was performed by the cheaper and sensitive NR uptake assay as previously described [14–16]. Samples were added in the culture medium so that dimethylsufoxide (DMSO) used prior for dilution, did not exceed 0.1% final concentration. Briefly, cells were detached by treatment with 0.25% trypsin/EDTA (Invitrogen, USA) and an aliquot of 1 **×** 10^4^ cells was placed in each well of a 96-well cell culture plate (Thermo Scientific, Germany) in a total volume of 200 μL. The cells were allowed to attach overnight and subsequently treated with different concentrations of the extracts and doxorubicin. Each of the studied samples were immediately added in varying concentrations in additional 100 μL of culture medium to obtain a total volume of 200 μL/well. After 72 h incubation in humidified 5% CO_2_ atmosphere at 37 °C, the medium was removed and 200 μL fresh medium containing 50 μg/mL NR was added to each well and incubation continued for an additional 3 h at 37 °C in 5% CO_2_ atmosphere. The dye medium was then removed and each well was then washed rapidly with 200 μL phosphate buffer saline (PBS) followed by addition of 200 μL of acetic acid-water-ethanol in water (1:49:50). The plates were kept for 15 min at room temperature to extract the dye and then shaken for a few minutes on a GFL 3012 shaker (Gesellschaft für Labortechnik mbH, Burgwedel, Germany). Absorbance was measured on ELx 808 Ultra Microplate Reader (Biotek) equipped with a 540 nm filter. Each assay was done at least three times, with three replicates each. The viability was evaluated based on a comparison with untreated cells. The IC_50_ values represented the sample’s concentrations required to inhibit 50% of cell proliferation and were calculated from a calibration curve by linear regression using Microsoft Excel [17].

### Flow cytometry for cell cycle analysis and detection of apoptotic cells

The cell-cycle analysis was performed by flow cytometry using BD cycletest™ Plus DNA Kit Assay (BD Biosciences, San Jose, USA). The BD Cycletest™ Plus DNA kit provides a set of reagents for isolating and staining cell nuclei. Flow cytometric analysis of differentially stained cells is used to estimate the DNA index (DI) and cell-cycle phase distributions. Briefly, MCF-7 cells (3 mL, 1 × 10^5^ cells/mL) were seeded into each well of 6-well plates and allowed to attach for 24 h. The cells which were treated with ¼ × IC_50_, ½ × IC_50_ and IC_50_ concentrations of *Elephantopus mollis* whole plant (EMW) and *Kalanchoe crenata* leaves (KCL) extracts and the standard drug, doxorubicin, and grown for 72 h. The untreated cells (control) were also included in the assay. They were further trypsinized and suspended in 1 mL PBS, then centrifuged at 400 g for 5 min at room temperature (RT). The cells were further processed according to the manufacturer’s protocol [16]. The cells were further measured on a BD FACS Aria I Cell Sorter Flow Cytometer (Becton-Dickinson, Germany). For each sample 10^4^ cells were counted. For PI excitation, an argon-ion laser emitting at 488 nm was used. Cytographs were analyzed using BD FACSDiva™ Flow Cytometry Software Version 6.1.2 (Becton-Dickinson).

### Caspase-Glo 3/7 and caspase-Glo 9 assay

Caspase activity in MCF-7 cells was detected using Caspase-Glo 3/7 and Caspase-Glo 9 Assay kits (Promega, Mannheim, Germany) as previously reported [18–20]. Cells were treated with EMW and KCL at their ½ × IC_50_ and IC_50_ values with DMSO as solvent control for 6 h. Luminescence was measured using an BioTek Synergy™ HT multi-detection microplate reader. Caspase activity was expressed as percentage of the untreated control.

### Analysis of mitochondrial membrane potential (MMP)

The MMP was analyzed in MCF-7 cells by 5,5′,6,6′-tetrachloro-1,1′,3,3′-tetraethylbenzimidazolylcarbocyanine iodide (JC-1; Biomol, Hamburg, Germany) staining as previously reported [18–20]. Cells (3 mL, 1 × 10^5^ cells/mL) treated for 72 h with different concentrations (¼ × IC_50_, ½ × IC_50_ and IC_50_) of EMW, KCL and doxorubicin (drug control) or DMSO (solvent control) were incubated with JC-1 staining solution for 30 min according to the manufacturer’s protocol, as earlier reported. Subsequently, cells were measured in a BD FACS Aria I Cell Sorter Flow Cytometer (Becton-Dickinson, Germany). The JC-1 signal was measured at an excitation of 561 nm (150 mW) and detected using a 586/15 nm band-pass filter. The signal was analyzed at 640 nm excitation (40 mW) and detected using a 730/45 nm bandpass filter. Cytographs were analyzed using BD FACSDiva™ Flow Cytometry Software Version 6.1.2 (Becton-Dickinson). All experiments were performed at least in triplicates.

### Measurement of reactive oxygen species (ROS)

The 2′,7′-dichlorodihydrofluorescein diacetate (H_2_DCFH-DA) (Sigma-Aldrich) was used for the detection of ROS in MCF-7 cells treated with EMW, KCL and doxorubicin (drug control) or DMSO (solvent control) using OxiSelect™ Intracellular ROS Assay Kit (Green Fluorescence) as recommended by the manufacturer (Cell Biolabs Inc., San Diego, USA). This is a cell-based assay for measuring hydroxyl, peroxyl, or other reactive oxygen species activity within a cell. The assay employs the cell-permeable fluorogenic probe 2′,7′-dichlorodihydrofluorescin diacetate (DCFH-DA). DCFH-DA is diffused into cells and is deacetylated by cellular esterases to non-fluorescent 2′,7′-dichlorodihydrofluorescin (DCFH), which is rapidly oxidized to highly fluorescent 2′,7′-dichlorofluorescein (DCF) by ROS. Cells (1 **×** 10^4^ cells) were treated with samples at ¼ × IC_50_, ½ × IC_50_ and IC_50_ for 24 h. After addition of 100 μL 1X DCFH-DA/DMEM solution to cells and incubation at 37 °C for 30–60 min, the fluorescence was measured using SpectraMax® M5 Microplate Reader (Molecular Devices, Biberach, Germany) at 480/530 nm. All experiments were performed at least in triplicates.

## Results

### Phytochemical composition of plants’ extracts

Table 1 displays the chemical composition of the extracts and reveals the presence of polyphenols, triterpenes and sterols in all extracts. Coumarins, flavonoids, alkaloids, saponins and tannins were selectively distributed.

### Cytotoxicity

The results of the antiproliferative activity of the tested extracts and doxorubicin as determined by the NR uptake assay are shown in Table 2. The selectivity index (Table 2) was determined as the ratio of IC_50_ value in the CRL2120 normal fibroblast, divided by the IC_50_ in the cancer cell line. Extracts EMW, KCL and doxorubicin had IC_50_ values below 40 μg/mL in the five studied carcinoma cell lines. The IC_50_ values of PCL were not detected at up to 40 μg/mL in all cancer cell lines whilst ECB, LAB and MML showed selective activities. The recorded IC_50_ values ranged from 3.29 μg/mL (towards DLD-1 colorectal adenocarcinoma cells) to 24.38 (against small lung cancer A549 cells) for EMW, from 2.33 μg/mL (mesothelioma SPC212 cells) to 28.96 μg/mL (HepG2 hepatocarcinoma) for KCL, and from 0.04 μg/mL (towards SPC212 cells) to 0.55 μg/mL (towards A549 cells) for doxorubicin. All extracts including the two most active ones (EMW and KCL) were less toxic towards normal CRL2120 fibroblast than carcinoma cells (selectivity indexes above 1.00) (Table 2). The best extracts, EMW and KCL as well as doxorubicin were further tested for the effects on cell cycle distribution, caspases activity, MMP loss and ROS production in MCF-7 cells.

**Table 2:** Cytotoxicity of tested plant extracts and doxorubicin towards cancer cell lines and normal cells as determined by the neutral red assay

Samples	Cell lines, IC_50_ values in μg/mL and selectivity index^a^ (in bracket)					
	A549	SPC212	HepG2	DLD-1	MCF-7	CRL2120
*Elephantopus mollis* (whole plant; EMW)	24.38 ± 1.86	**4.05 ± 0.69**	**3.74 ± 0.07**	**3.29 ± 0.04**	**3.97 ± 0.48**	>40
	(>1.64)	(>9.89)	(>10.70)	(>12.18)	(>10.08)	
*Enantia chlorantha* (bark; ECB)	>40	25.16 ± 1.30	**17.32 ± 0.13**	>40	>40	>40
		(>1.59)	(>12.31)			
*Kalanchoe crenata* (leaves; KCL)	**8.23 ± 0.15**(>4.86)	**2.33 ± 0.23**	28.96 ± 3.51	23.87 ± 1.69	**19.31 ± 0.79**	>40
		(>17.20)	(>1.38)	(>1.68)	(>2.07)	
*Lophira alata* (bark; LAB)	>40	>40	32.98 ± 4.20	>40	>40	>40
			(>1.21)			
*Millettia macrophylla* (leaves; MML)	>40	**7.54 ± 0.26**	**2.01 ± 0.04**	31.02 ± 2.86	25.99 ± 1.68	>40
		(>5.31)	(>19.90)	(>1.29)	(>1.54)	
*Phragmanthera capitata* (leaves; PCL)	>40	>40	>40	>40	>40	>40
Doxorubicin	**0.55 ± 0.11**	**0.04 ± 0.01**	**0.10 ± 0.01**	**0.20 ± 0.02**	**0.19 ± 0.03**	**0.32 ± 0.06**
	(0.58)	(8.63)	(3.18)	(1.58)	(1.67)	

^a^The selectivity index was determined as the ratio of IC_50_ value in the CRL2120 normal fibroblasts divided by the IC_50_ in the cancer cell lines. In bold: significant activity [7, 23, 24, 45]

### Mechanistic studies

**Fig. 1:**
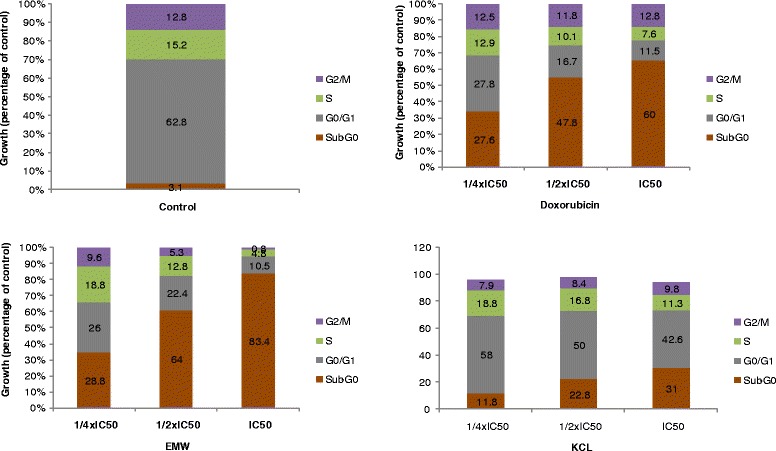
Effects of the extracts from the whole plant of *Elephantopus mollis* (EMW), *Kalanchoe crenata* leaves (KCL) and doxorubicin on cell cycle distribution in MCF-7 cells. IC_50_ values were 3.97 μg/mL (EMW), 19.31 μg/mL (KCL) and 0.32 μg/mL (doxorubicin)

**Fig. 2:**
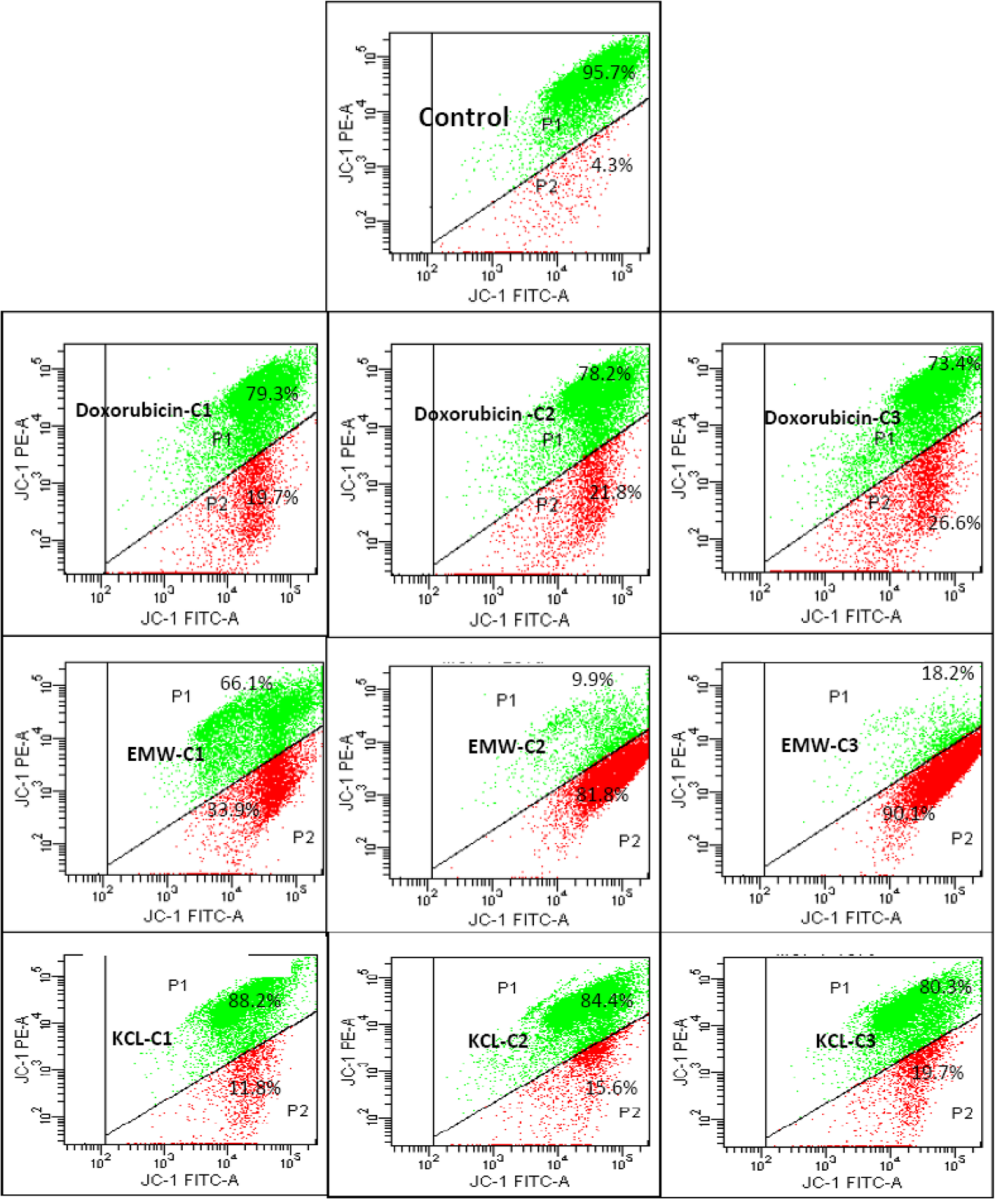
Effects of *Elephantopus mollis* whole plant (EMW) and *Kalanchoe crenata* leaves (KCL) extracts and doxorubicin on MMP in MCF-7 cells for 72 h. Cells were treated with ¼ × IC_50_ (C1), ½ × IC_50_ (C2) and IC_50_ (C3) of each compound. IC_50_ values were 3.97 μg/mL (EMW), 19.31 μg/mL (KCL) and 0.32 μg/mL (doxorubicin)

**Fig. 3:**
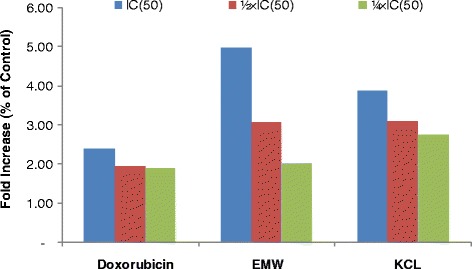
Effects of *Elephantopus mollis* whole plant (EMW) and *Kalanchoe crenata* leaves (KCL) extracts and doxorubicin for 24 h in ROS production in MCF-7 cells after treatment with. IC_50_ values were 3.97 μg/mL (EMW), 19.31 μg/mL (KCL) and 0.32 μg/mL (doxorubicin)

Cell cycle distribution in MCF-7 cells treated with EMM, KCL and doxorubicin is depicted in Fig. 1. EMW and KCL induced dose-dependent cell cycle modifications with progressive increase of sub-G0/G1 phase cells. Both EMW and KCL induced cell cycle arrest in G0/G1. Upon treatment of MCF-7 cells with the selected samples, they progressively underwent apoptosis; the increase of sub-G0/G1 cells ranged from 11.8% (¼ IC_50_) to 31% (IC_50_) for KCL, from 28.8% (¼ IC_50_) to 83.4% (IC_50_) for EMW, from 27.6% (¼ IC_50_) to 60% (IC_50_) for doxorubicin and only 3.1% in non-treated cells. Upon treatment of MCF-7 cells with EMW, KCL and doxorubicin with equivalent (eq.) to the ½ × IC_50_ and IC_50_ for 6 h, no activation of caspase 3/7 and caspase 9 activities was observed. MCF-7 cells were also treated with EMM, KCL and doxorubicin, and the integrity of the MMP was analyzed. Data shown in Fig. 2 indicate that treatments induced MMP loss, ranged from 33.9% at eq. to ¼ × IC_50_ to 90.1% at eq. to the IC_50_ for EMW, from 11.8% (¼ × IC_50_) to 19.7% (IC_50_) for KCL and 19.7% (¼ × IC_50_) to 26.6% (IC_50_) for doxorubicin. Upon treatment of MCF-7 cells with the selected at concentration eq. to ¼ × IC_50_, ½ × IC_50_ and IC_50_ values for 24 h, the production of ROS in cells was also analyzed (Fig. 3). EMW and KCL induced increased ROS levels of more than 3-folds (at IC_50_), as compared with non-treated cells whilst doxorubicin induced more than 2-folds increase.

## Discussion

Cancers appear as the leading cause of death globally, with 8.8 million deaths recorded in 2015. The most killing types are cancers of the lungs (1.69 million deaths), liver (788,000 deaths), colon (774,000 deaths), stomach (754,000 deaths) and breasts (571,000 deaths) [21]. In this work, we assessed the ability of six medicinal plants used in cancer treatment or disease states with symptoms related to cancer, to prevent the proliferation of various carcinoma cell lines, including lung, liver, colon and breast cancers. These investigated cancer types are amongst the worldwide leading cause of cancer death [21, 22]. Botanicals displaying IC_50_ values below 20 μg/mL have been said to be good cytotoxic samples [7, 23, 24]. IC_50_ values below 20 μg/mL were recorded with EMW, KCL, MML and ECB respectively in 4, 3, 2 and 1 of the 5 tested carcinoma cells. Importantly, IC_50_ values below 5 μg/mL were obtained with EMW in 4/5 carcinoma cell lines as well as KCL towards SPC212 cells and MML against HepG2 cells. These data highlight the usefulness of these extracts in the fight against solid cancers. This hypothesis is strengthened by the good selectivity index (SI > 1; Table 2) of the tested extract, which is compatible with their possible use in cancer chemotherapy.

*Elephantopus mollis* and *Millettia macrophylla* are traditionally used in the treatment of cancers [25, 26]. The two plants, Especially*e. mollis,* had cytotoxic effects on the tested carcinoma cells, validating their traditional use in the management of malignancies. In this study, plants used traditionally to treat disease states with symptoms related to cancer, were *Lophira alata*, *Enantia chlorantha*, *Phragmanthera capitata* and *Kalanchoe crenata*. Amongst them, only *P. capitata* was not active on the tested cancer cell lines. This also consolidates the recommandations that ethnopharmacological usages such as immune and skin disorders, inflammatory, infectious, parasitic and viral diseases should be taken into account when selecting plants that treat cancer [27].

To the best of our knowledge, the anticancer activity of *Enantia chlorantha, Lophira alata* and *Kalanchoe crenata* is being reported herein for the first time. The antiproliferative effect of ethyl acetate extract of *Elephantopus mollis,* collected from Penang Agriculture Department, Relau, Malaysia), on HepG2 cells, with the lowest IC_50_ value of 9.38 μg/mL, NCI-H23 cells (13.17 μg/mL), T-47D cells (12.57 μg/mL) and Caov-3 cells (42.11 μg/mL) [25, 28], was reported. A much more lower IC_50_ value of 3.74 μg/mL was obtained with samples from Cameroon. This could be explained by possible geographic variations in the chemical constitution of the plant. However, both studies confirm the cytotoxic potential of this plant. The cytotoxicity of methanolic extract and chalcone dimers from *L. alata* on Ehrlich Ascites carcinoma cells [29] was also reported in the present work. This plant was moderately active against HepG2 cells, providing additional information on the anticancer activity of the plant. The poor cytotoxic effects of compounds and phenolic fractions of *M. macrophylla* towards breast cancer cells MCF-7 and MDA-MB-231, was reported [26]. Data obtained herein are in accordance with this previous study, as a moderate effect of MML was obtained in MCF-7 cells. However, MML had good effect against SPC212 lung adenocarcinoma and HepG2 adenocarcinoma cells, highlighting its possible use in the fight against cancers.

Finally, evidences of the antiproliferative effects of the tested plant extract, highlights the good activity of *Elephantopus mollis, Kalanchoe crenata* and in lesser extent *Millettia macrophylla* have been provided*.* Extract of *E. mollis,* induced apoptosis in MCF-7 cells, mediated by MMP loss and increased ROS production whilst *Kalanchoe crenata* leaves extract induced apoptosis via ROS production (Figs. 2 and 3). It should be noted that only ROS production is not enough to identify cell apoptosis. Therefore, additional studies including detection of other molecules related to apoptosis such as BCL2, BAX, PRPP, etc., will be performed. Purification of the most active plants (*Elephantopus mollis, Kalanchoe crenata* and *Millettia macrophylla*) will also be performed to identify their cytotoxic constituents.

## Conclusions

In this work, the antiproliferative activity of extracts from six Cameroonian medicinal plants, *Lophira alata*, *Enantia chlorantha*, *Phragmanthera capitata*, *Kalanchoe crenata*, *Elephantopus mollis* and *Millettia macrophylla* was reported on five human solid cancer cell lines and normal CRL2120 fibroblasts. The three most active extracts were those from *E. mollis* whole plant, *K. crenata* leaves and *M. macrophylla* leaves. They can be used in the management of malignant diseases and deserve more exploration to isolate their active constituents in order to develop novel cytotoxic drugs.

## Abbreviations

ATCC: American Type Culture Collection
BAX: Bcl-2-associated X protein
Bcl-2: B-cell lymphoma 2
DCF: 2′,7′-dichlorofluorescein
DCFH: 2′,7′-dichlorodihydrofluorescin
DCFH-DA: 2′,7′-dichlorodihydrofluorescin diacetate
DMEM: Dulbecco’s Modified Eagle Medium
DMSO: Dimethylsufoxide
ECB: *Enantia chlorantha* bark extract
EDTA: Ethylenediaminetetraacetic acid
EMW: *Elephantopus mollis* extract
H_2_DCFH-DA: 2′,7′-dichlorodihydrofluorescein diacetate
HNC: Cameroon National Herbarium
KCL: *Kalanchoe crenata* leaves extract
LAB: *Lophira alata* bark extract
MML: *Millettia macrophylla* leaves extract
MMP: Mitochondrial membrane potential
NR: Neutral red
PARP: Poly(ADP-ribose) polymerase
PBS: Phosphate buffer saline
PCL: *Phragmanthera capitata* leaves extract
ROS: Reactive oxygen species
